# Papillary muscles in borderline left ventricules: morphological differences compared to normal

**DOI:** 10.1186/1532-429X-17-S1-P219

**Published:** 2015-02-03

**Authors:** María N Velasco Forte, Martin Grips, Mohamed S Nassar, Gerald F Greil, Tarique Hussain

**Affiliations:** Division of Imaging Sciencies and Biomedical Engineering, Guy’s and Saint Thomas Hospital, San José de La Rinconada, Spain; Paediatric Cardiology Unit, Virgen del Rocio Hospital, Seville, Spain; King’s College London, London, UK

## Background

In healthy subjects the papillary muscles (PM's) are usually organized as two equal-sized muscles holding an anterolateral and posteromedial position in the left ventricle (LV). However, as described in observational autopsy studies, their morphology may vary. There may be two clear PM's or one or both muscles may be split into several groups. In this study, morphological differences in the PM's in borderline and normal-sized LV's were assessed using CMR.

## Methods

Children undergoing CMR for clinical reasons were included in the study. This comprised of thirty-three consecutive borderline LV cases and twenty-nine consecutive subjects with a normal-sized LV. The morphology of the PM's, their location and the angle created between them was assessed using a steady-state-free-precession short-axis (SAx) cine and 3D-whole-heart sequences. IRB approval was obtained for retrospective analysis.

## Results

The median age was 4.3 years (range: 4months to 20 years; 35 male patients).The combination of SAx cine imaging and 3D-whole-heart imaging demonstrated PM anatomy in all cases.

All patients with normal-sized LV had two major muscle groups whereas 33% of children with borderline LV only had a single PM (Pearson X^2^:p=0.001). When a single PM was observed, it held a posteromedial position in 82% of the cases. However, there was no significant difference between the location of the PM support between both groups.

Splitting of PM into groups was found regularly in both LV types. In borderline LV, groups were often split widely at their insertion to the LV giving a broad-based pedicle (33% of cases, Figure 1). However, a narrow or fused pedicle was always observed in normal size LV.

The mean angle between PM insertion points was similar between groups (1120 (SD 150) in normal LV and 1260 (SD 310) for borderline ones when two narrow pedicles (n=14) were observed).

Eight children underwent successful biventricular repair in this series of borderline LV. Seven of these had two narrow/fused pedicles (X^2^:p=0.006 versus group undergoing univentricular repair).

## Conclusions

In normal-sized LV, the PM's are dual with narrow/fused pedicles. In the hypoplastic/borderline LV, there is often only a single PM and the pedicle of the PM's is often split with a broad base. Having two PM's with narrow/fused origins appears to be an additional indicator of favourable anatomy for biventricular repair. A combination of 3D whole-heart and short-axis cine is required to assess papillary morphology accurately.

